# Cognitively-Related Basic Activities of Daily Living Impairment Greatly Increases the Risk of Death in Alzheimers Disease

**DOI:** 10.1371/journal.pone.0160671

**Published:** 2016-08-29

**Authors:** Fu-Wen Liang, Wenyaw Chan, Ping-Jen Chen, Carissa Zimmerman, Stephen Waring, Rachelle Doody

**Affiliations:** 1 Department of Public Health, National Cheng Kung University Hospital, College of Medicine, National Cheng Kung University, No.1, University Road, Tainan City 701, Taiwan; 2 Department of Biostatistics, University of Texas-Health Science Center at Houston, 1200 Pressler Street, E827, Houston, Texas 77030, United States of America; 3 Department of Geriatrics and Gerontology, Chi-Mei Medical Center, 901, Zhong-Hua Rd., Yong-Kang Dist., Tainan City 710, Taiwan; 4 Department of Psychology, Rice University, 6100 Main MS-27, Houston, Texas 77005, United States of America; 5 Essentia Institute of Rural Health, 502 East Second Street, Duluth, MN 55805, United States of America; 6 Alzheimer's Disease and Memory Disorders Center, Baylor College of Medicine,7200 Cambridge Street, A9.210, Houston, Texas 77030, United States of America; University Of São Paulo, BRAZIL

## Abstract

**Introduction:**

Some Alzheimer’s disease (AD) patients die without ever developing cognitively impaired basic activities of daily living (basic ADL), which may reflect slower disease progression or better compensatory mechanisms. Although impaired basic ADL is related to disease severity, it may exert an independent risk for death. This study examined the association between impaired basic ADL and survival of AD patients, and proposed a multistate approach for modeling the time to death for patients who demonstrate different patterns of progression of AD that do or do not include basic ADL impairment.

**Methods:**

1029 patients with probable AD at the Baylor College of Medicine Alzheimer’s Disease and Memory Disorders Center met the criteria for this study. Two complementary definitions were used to define development of basic ADL impairment using the Physical Self-Maintenance Scale score. A weighted Cox regression model, including a time-dependent covariate (development of basic ADL impairment), and a multistate survival model were applied to examine the effect of basic ADL impairment on survival.

**Results:**

As expected decreased ability to perform basic ADL at baseline, age at initial visit, years of education, and sex were all associated with significantly higher mortality risk. In those unimpaired at baseline, the development of basic ADL impairment was also associated with a much greater risk of death (hazard ratios 1.77–4.06) over and above the risk conferred by loss of MMSE points. A multi-state Cox model, controlling for those other variables quantified the substantive increase in hazard ratios for death conferred by the development of basic ADL impairment by two definitions and can be applied to calculate the short term risk of mortality in individual patients.

**Conclusions:**

The current study demonstrates that the presence of basic ADL impairment or the development of such impairments are important predictors of death in AD patients, regardless of severity.

## Introduction

Alzheimer’s disease (AD) is a progressive neurodegenerative disorder that impairs cognition and daily functioning. Affected patients all die with AD, although the factors that predict survival overlap with those that predict survival in non-demented individuals of similar age: especially age, education, and comorbid conditions [1–11]. Strong negative associations between survival and measures of AD symptom severity would suggest that simple disease progression may drive the duration of survival with AD [1,3,5–10]. Yet, the observation that some patients do and some patients do not pass through a stage of impaired basic ADL prior to death weakens this particular view.

If the development of basic ADL impairment exerts an effect on survival that is separate from disease severity, estimating the time to death for patients with and without such basic ADL impairment could be helpful in planning effectively for future demands on medical and social resources [12]. Specifically, patients with impaired basic ADL may require higher care costs in the short term but lower projected medical costs compared to a less impaired subject if they die sooner. The purpose of our study was to examine the relationship between the development of basic ADL impairment, in models that include known predictors of survival, and risk of death among AD patients. Using multistate modeling, we provide a natural way to handle the transitions between disease onset, development of basic ADL impairment, and death while controlling for the expected effects of other baseline characteristics on survival.

## Materials and Methods

### Ethics statement

Patients came to the Baylor College of Medicine Alzheimer’s Disease and Memory Disorders Center (ADMDC) between January 1989 and February 2008 were eligible to participate in the ADMDC longitudinal follow-up database [13]. This study was approved by the Baylor College of Medicine Institutional Review Board (H-9095). Written informed consent was obtained from all participants.

### Patient identification

Of 1484 probable AD patients (NINCDS-ADRDA [14], 1029 (69%) met inclusion criteria for this study (i.e., had at least one follow-up visit and lived in the greater Houston area at the time of initial visit). Sociodemographic factors (age, sex, race, marital status, and education level), comorbid condition, and a standardized estimate of duration of cognitive symptoms were obtained at baseline along with a battery of psychometric tests as previously described [13,15]. Using the National Cholesterol Education Program–Adult Treatment Panel III guidelines, a cardiovascular disease equipment (CVDE) [16] was calculated based on the history of myocardial infarction, congestive heart failure, stent placement, diabetes mellitus, or high risk for congestive heart disease with any two of hypertension, hyperlipidemia, or current cigarette smoking. Patients were reassessed annually with the same battery of tests. If patients could not be contacted by phone after three attempts, they were considered lost to follow-up. Vital status was obtained from the National Death Index at six month intervals.

### Measures

The duration of AD symptoms was estimated by an ADMDC physician using a standardized procedure [15]. The Mini Mental Status Examination (MMSE) was used to measure dementia severity [17]. Cognitively related basic ADL were assessed with the Physical Self-Maintenance Scale (PSMS) questionnaire developed by Lawton and Brody [18]. Six basic functional abilities were surveyed: bowel and bladder control, feeding, dressing, grooming, ambulation, and bathing. All six items were rated by discussion between a psychometrician and a caregiver on a 5-point rating scale from 1 (no impairment) to 5 (severe impairment) with descriptors for each level of severity. Our psychometricians are trained to rate the cognitive contribution to basic ADL impairment and to disregard impairment related to a physical comorbidity e.g. post-transurethral prostatectomy urinary incontinence. A total score of 6 represents completely intact basic ADL and scores greater than 6 at baseline were considered impaired. Two different definitions were utilized to define the development of basic ADL impairment after baseline: 1) a PSMS score greater than 6 at a post-baseline visit; or 2) an increase in PSMS score of ≥ 2 points relative to baseline. These two complementary definitions allowed for the assessment of both the new development of basic ADL difficulty and the relative worsening of basic ADL function compared to a patient's own baseline score.

### Statistical analysis

First, descriptive statistics of the demographic and clinical characteristics were generated, and comparisons between the two groups of interest (patients with intact ability to perform basic ADL and those with impaired ability to perform basic ADL) were performed using either the *χ*^2^ test, the Student’s t test, or the log-rank test. Second, we used fixed-covariate Cox modeling to examine the simultaneous effects of potential independent variables on survival. The validity of the proportional hazards assumption was assessed using Schoenfeld residuals for each variable of interest. Third, time-dependent covariates, namely, development of basic ADL impairment (intact versus impaired), were added to the final Cox model to evaluate their corresponding effects on survival, adjusting for the effects of other selected variables. In addition, the inverse probability of censoring weights (IPCW) [19] was used as weighting in the Cox regression to adjust for possible bias due to loss to follow-up. The estimated weights are based on the predicted probability generated from a logistic regression in which the response variable tells whether the patient was lost to follow-up or not and the independent variables include age at first visit, sex, race, marital status, and education level, baseline PSMS and MMSE scores, duration of cognitive symptoms, and CVDE.

**Fig 1:**
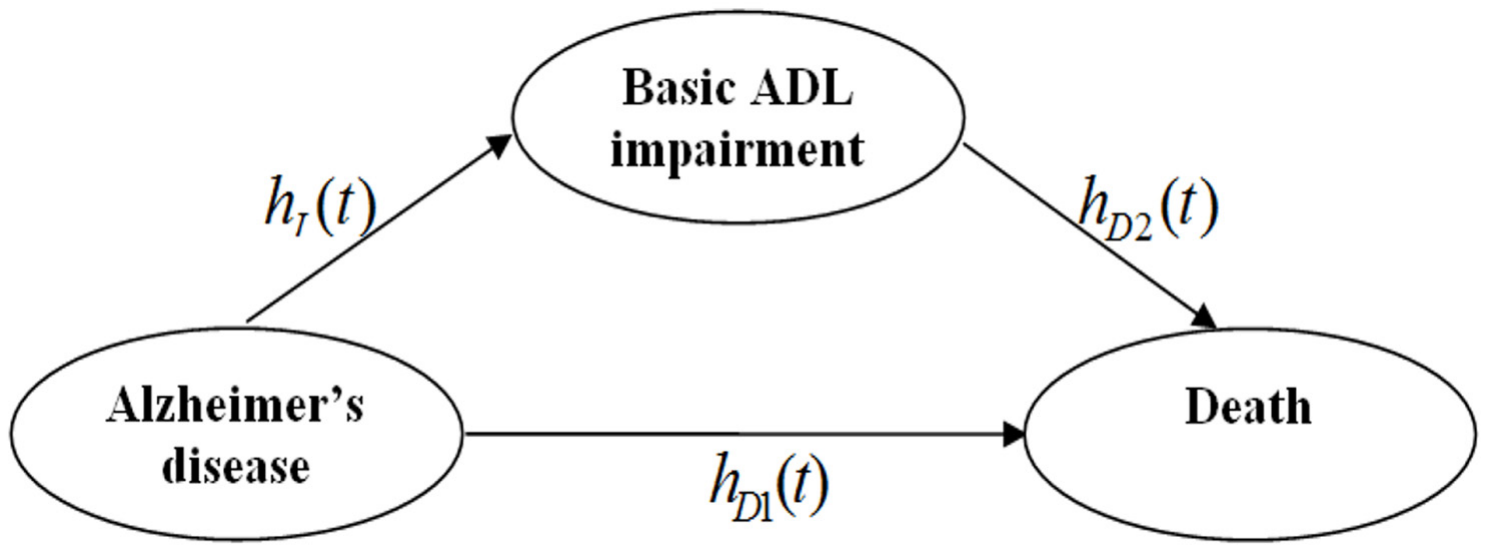
Three-state Markov chain used to derive predictive value of basic ADL impairment. *h*_*I*_*(t)* is the hazard rate for the time to basic ADL impairment. *h*_*D*1_*(t)* is the hazard rate for the time to death among basic ADL-intact patients. *h*_*D*2_*(t)* is the hazard rate for the time to death among basic ADL-impaired patients. The transitions between disease onset, development of basic ADL impairment, and death were modeled by quantifying the effect of baseline characteristics on basic ADL impairment and survival as well as the effect of developing basic ADL impairment on survival.

Finally, the effect of change in basic ADL status on the prediction of patient outcome was examined using a novel application of multistate survival analysis methods [20–22]. In this approach, the development of Alzheimer’s disease, onset of basic ADL impairment, and death were treated as a three-state Markov chain (Fig 1), and the transitions between phases were modeled. We established a separate overall death rate for those without basic ADL impairment (any individual having impaired basic ADL prior to death was censored to determine this rate, Fig 1, hD1(t)), as well an overall death rate for those who had or developed basic ADL impairment (we used a left-truncated likelihood such that patients entered the risk set at the onset of basic ADL impairment to determine this rate, Fig 1, hD2(t)). A Cox model was then fitted to the data, using the occurrence of basic ADL impairment as the event. Cumulative hazard functions were calculated using the estimates of the risk coefficients and the cumulative baseline hazards using Breslow’s approach [23]. By applying this multistate approach, we obtained a complete picture of how the change in a patient's basic ADL impairment status influences the prediction of his or her survival in the presence of important covariates, that could differ for any path in the Markov chain. All statistical analyses were performed using SAS version 9.1 (SAS Inc., Cary, NC). All tests were considered significant at the 0.05 level.

## Results

Baseline characteristics of the 1029 patients in our sample are shown in Table 1. Among them, 552 were censored at the end of the study period or at the time of last known contact (N = 176). The majority of participants were white, female, and married. Compared to patients who had intact basic ADL, patients who had impaired basic ADL at baseline were significantly older, less educated, and had a longer duration of AD symptoms but the percentage of females did not differ in the two groups. As predicted, median survival time was significantly shorter (by almost 3 years) for patients who reported basic ADL impairment at the first visit (p<0.0001). Furthermore, 45.4% of patients with basic ADL impairment survived compared to 64.0% of patients who were not initially impaired (p<0.0001).

**Table 1:** Baseline patient characteristics and corresponding comparisons by basic activities of daily living (basic ADL) impairment status.

Characteristics	All patients (n = 1029)	Basic ADL-intact at baseline (n = 458)	Basic ADL-impaired at baseline (n = 571)	p-value
Female, n (%)	708 (68.8)	306 (66.8)	402 (70.4)	0.224
White, n (%)	935 (90.9)	436 (95.2)	499 (87.4)	<.0001^¥^
Married, n (%)	626 (60.8)	315 (68.8)	311 (54.5)	<.0001^¥^
Death, n (%)	477 (46.4)	165 (36.0)	312 (54.6)	<.0001^¥^
CVDE^$^, n (%)	632 (62.2)	271 (59.6)	361 (64.4)	0.118
Age at baseline*	75.1 ± 8.3	72.7 ± 8.1 (39, 92.8)	77.1 ± 8.0 (46.2, 96.5)	<.0001^€^
Formal education*	13.6 ± 3.6	14.3 ± 3.5 (2, 29)	13.1 ± 3.6 (0, 26)	<.0001^€^
Symptom duration*	3.9 ± 2.3	3.4 ± 2.1 (0.5, 18)	4.3 ± 2.4 (0.5, 13)	<.0001^€^
MMSE score at baseline*	19.0 ± 7.0	22.4 ± 4.9 (0, 30)	16.3 ± 7.3 (0, 30)	<.0001^€^
PSMS score at baseline*	8.5 ± 3.8	6.0 (6, 6)	10.5 ± 4.1 (7, 28)	<.0001^€^
Median survival from initial visit (years)	6.6	8.1	5.3	<.0001^£^

* Results are reported as means ± SD (maximum, minimum) for continuous variables.

^€^ Student’s *t* test, p < 0.001;

^¥^*χ*^2^ test, *p* < 0.001;

^£^log-rank test, *p* < 0.001.

All statistical results refer to the comparison of the basic ADL-intact versus the basic ADL-impaired groups.

^$^ CVDE: Cardiovascular disease equipment

In the weighted Cox regression model (Table 2), age, education, sex, and baseline PSMS and MMSE scores were significant predictors of survival whereas race, marital status, and duration of dementia symptoms had no significant univariate effects on survival. Not unexpectedly, older age was associated with shorter mean survival times, as were less education and male sex when other variables in the multiple model were held constant. Also consistent with past literature, higher scores on the MMSE were associated with longer survival; for every one point reduction on the baseline MMSE, there was a 4% increase in adjusted risk of death. Higher baseline PSMS scores (reflecting basic ADL impairment) were associated with a higher risk of death, even when we controlled for the other demographic and clinical variables: For every one-point increase in baseline PSMS score, there was a 6% increase in risk of death, even when controlling for the other variables. This finding supports our hypothesis that basic ADL impairment may be an independent predictor of survival beyond disease severity alone.

**Table 2:** Hazard ratios (95% confidence intervals) in the baseline Cox regression model and time-dependent Cox regression models for death.

	Baseline Model	Time-dependent Model for Death	
Variable		PSMS ≥ 7	ΔPSMS ≥ 2^a^
Age at baseline	1.03 (1.02, 1.04)	1.03 (1.01, 1.04)	1.03 (1.02, 1.04)
Sex (Ref: Female)	1.63 (1.41, 1.89)	2.02 (1.62, 2.51)	1.55 (1.34, 1.79)
Race (Ref: White)	0.82 (0.65, 1.04)	0.31 (0.18, 0.51)	0.92 (0.73, 1.15)
Education (years)	0.97 (0.96, 0.99)	0.97 (0.95, 0.99)	0.97 (0.97, 0.99)
MMSE at baseline	0.96 (0.95, 0.97)	0.94 (0.92, 0.96)	0.97 (0.96, 0.98)
PSMS at baseline	1.06 (1.04, 1.08)	---	---
Symptom duration	1.00 (0.97, 1.03)	0.99 (0.94, 1.04)	1.01 (0.98, 1.04)
Marital status(Ref: Married)	0.97 (0.84, 1.11)	0.72 (0.57, 0.90)	0.91 (0.79, 1.05)
CVDE^$^	1.10 (0.96, 1.25)	1.04 (0.85, 1.26)	1.09 (0.96, 1.24)
ADL impairment during follow-up	---	1.77 (1.41, 2.22)	4.06 (3.30, 5.01)

^a^An increase in PSMS score of **≥** 2 points relative to baseline

^$^ CVDE: Cardiovascular disease equipment

Of the 458 patients whose basic ADL were not impaired at baseline 50.9% (N = 233) developed such impairment by the first definition (PSMS ≥ 7) and 41.5% (N = 190) by the second definition (gained 2 or more points). Because the baseline model does not quantify the effect of developing basic ADL impairment on survival over time, we looked at a time-dependent model. The results of the Cox regression analysis including time-dependent variables (the development of basic ADL impairment) are also displayed in Table 2. The development of basic ADL impairment, defined by development of a PSMS score greater than 6, was strongly and significantly associated with an increased risk of death relative to no basic ADL impairment (hazard ratio 1.77). Age, education and baseline MMSE score had modest but significant effects on survival in this model. There was also the expected effect of male sex reducing survival, but with a lower hazard ratio (1.54) compared to the baseline model predictive value. This argues for the need to include the time-dependent variable. Using our second definition of basic ADL impairment (increase of 2 or more points on the PSMS post-baseline), the effect of worsening basic ADL impairment was even strong and significantly associated with mortality (hazard ratio 4.06), while age, education, baseline MMSE, and sex had similar predictive values to the model using the first definition. The results in Table 2 suggest that the impact of ADL impairment on mortality is at least as strong as the impact of sex, and stronger than the impact of disease severity.

Finally, we used a novel multistate modeling approach to estimate the appropriate coefficients or contributions of each variable to the risk of death. We included all potential predictors from the baseline model (age at first visit, sex, race, education level, baseline MMSE score, symptom duration, marital status, and CVDE). We set up a multi-state Cox model defining one state as pre-basic ADL impairment and one state as post ADL impairment. First we designated basic ADL impairment as the outcome event and, the best-fit model revealed that only age at first visit, baseline PSMS and MMSE scores were significant predictors of time to impairment, when controlling for other relevant factors (Table 3). We then moved to a two-stage model selecting survival as the outcome event and applied the estimates from the multistate modeling approach to simultaneously predict the probability of death for an average patient before and after basic ADL impairment (Table 3). The specific hazard ratios in Table 3 allow for individual prediction of mortality within a set interval of time.

**Table 3:** Hazard ratios (95% confidence intervals) in the multistate model for predicting basic ADL impairment or death.

	Risk of Basic ADL impairment		Risk of Death before basic ADL impairment		Risk of Death after basic ADL impairment	
Variable	PSMS ≥ 7	ΔPSMS ≥ 2^a^	PSMS ≥ 7	ΔPSMS ≥ 2^a^	PSMS ≥ 7	ΔPSMS ≥ 2^a^
Age at baseline	1.01 (0.99, 1.02)	1.01 (1.00, 1.02)	1.05 (1.03, 1.08)	1.04 (1.03, 1.05)	1.01 (0.99, 1.03)	1.02 (1.01, 1.04)
Sex (Male:Female)	0.97 (0.82, 1.15)	0.99 (0.85, 1.17)	2.08 (1.39, 3.12)	1.83 (1.50, 2.23)	1.92 (1.47, 2.52)	1.26 (1.01, 1.58)
Race	1.04 (0.80, 1.34)	1.21 (0.93, 1.59)	1.36 (0.72, 2.56)	1.28 (0.94, 1.73)	12.5 (4.16, 37.8)	0.87 (0.60, 1.25)
Education	1.02 (1.00, 1.04)	1.01 (0.99, 1.03)	0.95 (0.90, 1.01)	0.94 (0.92, 0.97)	0.99 (0.95, 1.02)	1.01 (0.98, 1.03)
MMSE at baseline	0.93 (0.91, 0.95)	0.95 (0.94, 0.96)	0.91 (0.88, 0.96)	0.94 (0.92, 0.95)	0.94 (0.92, 0.97)	0.98 (0.96, 0.99)
Symptom duration	0.99 (0.96, 1.03)	1.00 (0.97, 1.03)	0.97 (0.89, 1.05)	1.02 (0.98, 1.06)	1.02 (0.95, 1.09)	1.01 (0.96, 1.05)
Marital status	0.90 (0.77, 1.06)	0.98 (0.84, 1.14)	0.70 (0.47, 1.06)	0.83 (0.69, 1.01)	1.85 (1.41, 2.43)	1.32 (1.07, 1.64)
CVDE^$^	1.07 (0.92, 1.25)	1.03 (0.90, 1.19)	0.59 (0.41, 0.84)	1.08 (0.90, 1.29)	1.36 (1.07, 1.72)	1.10 (0.91, 1.33)

^a^ΔPSMS = An increase in PSMS score of **≥** 2 points relative to baseline

^$^ CVDE: Cardiovascular disease equipment

**Fig 2:**
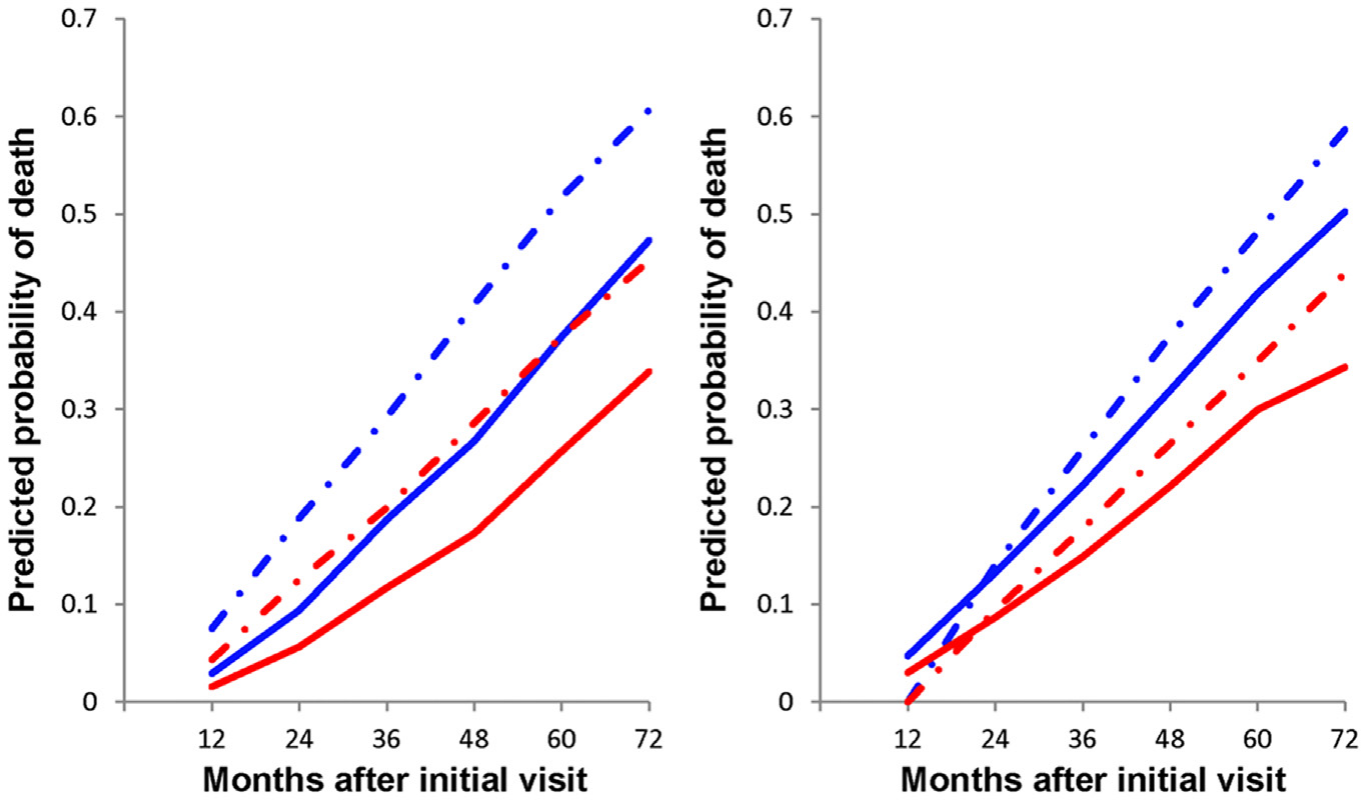
Predicted conditional probability of death for patients with and without cognitive based basic ADL impairment. (A)In the left panel, impairment was defined by PSMS ≥ 7. (B)In the right panel, impairment was defined by PSMS ≥ 2 points of increase relative to baseline. The predicted conditional probability of death over the first 6 years was calculated for each sex (men in blue and women in red) assuming either intact (the solid lines) or impaired (the dashed lines) basic ADL at time of observation.

In order to demonstrate the value of the multi-state model, we generated survival curves for male and female example patients. ADMDC population information was used to determine the characteristics of an average patient: 75.1 years old at the initial clinic visit with 13.6 years of education and a baseline MMSE score of 19.0. We used the estimates from Table 3 to graph the predicted conditional probability of death in the first 6 years (the overall median survival time of our population was 6.6 years) for a patient who did (the dashed lines) or did not (the solid lines) develop basic ADL impairment over the period of observation (Fig 2). Because sex was also a significant factor in predicting survival time, we calculated the conditional probability of death for each sex separately (men in blue and women in red). As illustrated in Fig 2, the development of basic ADL impairment predicts a much greater chance of death for both sexes. The effect of impaired ADL was most notable with the stricter definition of basic ADL impairment consisting of PSMS score greater than normal (>6). Male patients who developed basic ADL impairment had the greatest predicted probability of death, whereas female patients with no impairment showed the lowest predicted probability of death. The risk of death for impaired women was close to the risk for unimpaired men. The figure illustrates the relative risks of mortality for comparison purpose.

## Discussion

The current study is the first to demonstrate a strong relationship between either the presence or development of basic ADL impairment and the risk of death among AD patients, and the first to quantify this risk over a relevant clinical interval (6 years). The development of basic ADL impairment was a strong signal of increased likelihood of death in both men and women, surpassing the importance of the known risk factors of age, sex, and baseline cognitive scores. This increase in risk was significant and substantial even when disease severity was controlled for in the analysis. Our data can be used to provide quantitative estimates of how changes in an individual patient’s functional abilities will affect their predicted survival over a 6-year period.

Other results used in our model were consistent with previous studies showing that male sex, older age, less education, lower baseline MMSE score, and lower baseline basic ADL function significantly increase the risk of death in patients with AD [1–11,24–28]. Importantly, our analysis extends an understanding of the factors associated with mortality by showing that the development of basic ADL impairment is a harbinger of mortality, even when controlling for these other factors. For each point of basic ADL impairment at baseline, the risk of mortality increases 6%—which was comparable to the effects of age, education and baseline MMSE, and less than the effect of sex. However, the new development of basic ADL impairment, defined as either any increase in score or addition of two points of worsening was associated with a much greater risk of death: 1.77 times the risk by the definition of PSMS score greater than 6, and 4.06 times higher based upon a definition of gaining two or more points on this measure. This finding of an increased risk of mortality among patients who developed the basic ADL impairment during the follow-up further supports the importance of basic ADL impairment, regardless of timing of occurrence. Gambassi *et al* who investigated residents newly admitted to nursing homes with a diagnosis of probable Alzheimer’s disease found that the baseline basic ADL impairment was associated with increased mortality. However, Aguero-Torres *et al* found the impaired baseline functional status was associated with decreased survival in demented adults, but it became insignificant in patients with Alzheimer’s disease.

Researchers have not reached a consensus regarding the best methods for modeling the progression of Alzheimer’s disease [29,30]. To best model the occurrence and timing of an intermediate event (in our case, onset of basic ADL impairment) that precedes the event of interest (in our case, death), Klein et al. [21] suggested using a Cox model with time-dependent covariates, whereas Andersen et al. [20] proposed modeling distinct baseline hazard rates for each possible transition in the multistate process. One common concern in such studies is the potential for biased hazard ratio estimates when between-group comparisons are done using baseline values and without considering the events that occur between baseline and death; such events may have the same effect in both groups of patients, have different effects in each group, or change the rate of death in one group while having no effect in the other. Even when differences in survival are found, the timing of intermediate events could mask differences in marginal probabilities of death between patient groups or make differences appear to exist when there are none. We addressed these issues in two ways: 1) by using time-dependent basic ADL impairment in a Cox regression model and 2) by using a multistate survival model in which a combination of proportional hazards regression and left-truncated proportional hazards regression were applied to synthesize estimates of predictive probabilities of death while taking into consideration the development of basic ADL impairment over time. We demonstrated that the second model is more accurate than the first model because it takes into consideration more than one path of progression and allows predictions to be made about individuals whose characteristics vary.

One advantage of the current study is that it included a larger cohort of AD patients than many other studies. In fact, the participants in the Baylor ADMDC cohort represent one of the largest samples of individuals diagnosed with AD by standardized criteria and followed longitudinally in the United States. Another strength of our study was that only cases diagnosed as probable AD by NINCDS-ADRDA criteria [14] were used, which ensured a more homogeneous sample. Additionally, vital status information was available for 100% of the patients, which is another strength.

Despite the benefits of our large sample size, this particular cohort does have some limitations. The Baylor College of Medicine ADMDC is a specialty center associated with an academic institution. The sample used in this non-population-based study may contain a disproportionate number of well-informed participants who have better access to health care and fewer comorbid conditions (and therefore higher rates of survival) than the general population [31]. However, this concern is mitigated by the fact that we accept all patients, whether self-referred or referred by others, and are one of the few memory disorders and dementia clinics in the southwestern United States. A limitation of our study is the fact that measurement of basic ADL impairment can be confounded by the presence of comorbid conditions affecting physical function but not cognition. To avoid this, our psychometricians are trained to rate the items on the PSMS based upon cognitive causes rather than physical causes after discussion with the caregiver. Some judgment is still involved in making this assessment, which is a limitation. It is also possible that some overestimation of true survival occurred because patients with rapidly progressing AD who died before they could be diagnosed were not included in our sample. We did not enter item-level data on the basic ADLs, so we cannot assess their significance for survival individually. Finally, comorbid conditions, such as cardiovascular disease, diabetes mellitus, and chronic obstructive pulmonary disease, were not simultaneously analyzed in the current study, because they did not play a significant role in patient survival in other analyses of our population [31,32].

## Conclusions

There is a relative lack of data in the literature regarding longitudinal aspects of AD. It is essential to determine which factors contribute to AD patients’ risk of death so that preventive and protective measures may be implemented and to improve planning for resources over the course of the disease. Such analyses have clear implications for health economic analysis and public policy. The current study demonstrates that both relative and absolute impairment in basic ADL are important predictors of death in patients diagnosed with AD even when controlling for other factors that influence survival. Our method can quantify this increased risk of death in individual patients when they develop such impairment. In the future, we hope to explore how AD treatment influences the likelihood of developing basic ADL impairment at any given time, as well as the survival of patients who have already developed such impairment.

## Supporting Information

S1 File Data file. (XLSX)
